# Detection of plant cadmium toxicity by monitoring dielectric response of intact root systems on a fine timescale

**DOI:** 10.1007/s11356-024-33279-w

**Published:** 2024-04-12

**Authors:** Imre Cseresnyés, Tünde Takács, Anna Füzy

**Affiliations:** Institute for Soil Sciences, HUN-REN Centre for Agricultural Research, Herman Ottó Út 15, 1022 Budapest, Hungary

**Keywords:** Electrical capacitance, Dissipation factor, *In situ* root methods, Membrane permeability, Root conductance, Stomatal conductance, Transpiration, Water uptake rate

## Abstract

The root dielectric response was measured on a minute scale to assess its efficiency for monitoring short-term cadmium (Cd) toxicity non-destructively. Electrical capacitance (C_R_), dissipation factor (D_R_) and electrical conductance (G_R_) were detected during the 24 to 168 h after Cd treatment (0, 20, 50 mg Cd^2+^ kg^–1^ substrate) in potted maize, cucumber and pea. Stress was also evaluated by measuring leaf chlorophyll content, F_v_/F_m_ and stomatal conductance (g_s_) *in situ*, and shoot and root mass and total root length after harvest. C_R_ showed a clear diurnal pattern, reflecting the water uptake rate, and decreased significantly in response to excessive Cd due to impeded root growth, the reduced tissue permittivity caused by accelerated lignification, and root ageing. Cd exposure markedly increased D_R_, indicating greater conductive energy loss due to oxidative membrane damage and enhanced electrolyte leakage. G_R_, which was coupled with root hydraulic conductance and varied diurnally, was increased transiently by Cd toxicity due to enhanced membrane permeability, but declined thereafter owing to stress-induced leaf senescence and transpiration loss. The time series of impedance components indicated the comparatively high Cd tolerance of the applied maize and the sensitivity of pea cultivar, which was confirmed by visible shoot symptoms, repeated physiological investigations and biomass measurements. The results demonstrated the potential of single-frequency dielectric measurements to follow certain aspects of the stress response of different species on a fine timescale without plant injury. The approach can be combined with widely used plant physiological methods and could contribute to breeding crop genotypes with improved stress tolerance.

**Supplementary Information:**

The online version contains supplementary material available at 10.1007/s11356-024-33279-w.

## Introduction

In the plow-layer of agricultural soils, cadmium (Cd) usually ranges from 0.1 to 1 mg kg^–1^ worldwide, with mean of 0.4 mg kg^–1^, and rarely exceeds the 1–5 mg kg^–1^ maximum allowable concentration (McLaughlin et al. 2021). However, Cd concentration locally increases up to 10–40 mg kg^–1^ from geogenic sources, even reaches hundreds of mg kg^–1^ due to anthropogenic topsoil contamination (Kubier et al. 2019). Toxic levels of Cd accumulation in soils due to human activities, *e.g*. urban traffic, mining, industrial wastes, sewage sludge, phosphate fertilizers or agrochemicals, became a global problem (Bali et al. 2020). Cd is a non-essential heavy metal, and the highly soluble Cd^2+^ is taken up rapidly by plants and transferred to the food chain, threatening human health. The partitioning and mobility of Cd is regulated by the pH, colloidal and organic matter composition, cation exchange capacity and microbial activity of the soil (Bali and Sidhu 2022). Soil acidification induces Cd desorption from the binding sites, leading to enhanced availability of Cd^2+^ to plants. Cd excess induces several biochemical and physiological changes in plants, including disturbed water and nutrient uptake and transport, protein denaturation and reduced enzyme activity, oxidative damage caused by the generation of reactive oxygen species (ROS), chloroplast degradation, inhibited chlorophyll (Chl) biosynthesis and photochemical reactions, and decreased stomatal conductance (g_s_) and transpiration rate (Rizwan et al. 2016; Sahoo et al. 2022). These, in turn, can lead to visible toxicity symptoms, such as wilting, leaf roll, chlorosis and necrosis, as well as reduced organ growth, biomass and grain production. In general, Cd ions are mainly accumulated in the roots, where they impair membrane integrity (Pavlovkin et al. 2006), damage root tips, restrict root growth, and alter root anatomy (Lux et al. 2011). Root response is critical to plant stress tolerance, which varies with species, genotype, and growing conditions, and depends on the level, timing and duration of Cd exposure (Bali et al. 2020). Monitoring the root growth and function under heavy metal excess could facilitate the evaluation of plant sensitivity to Cd, and thus the identification of more tolerant crop cultivars (Akhtar et al. 2017). For this purpose, the application of dynamic, non-intrusive root methods, including electrical techniques, is preferable (Liu et al. 2021).

The polarization of root membranes changes the amplitude and phase of the input alternating current (AC) signal, and generates a measurable impedance response (Ehosioke et al. 2020). Thus, roots are considered analogous to cylindrical capacitors, in which the membranes, as dielectrics, store electric charges (Dalton 1995). The resultant capacitance (C) is directly proportional to the surface area (A) and the relative permittivity (*ε*_r_), and inversely proportional to the thickness (d) of the membranes: C = *ε*_0_ × *ε*_r_ × A × d^–1^, where *ε*_0_ is the vacuum permittivity. In plant materials, electrical polarization occurs concurrently with electrical conduction: roots are leaky capacitors with high energy losses. They are equivalent to parallel resistance–capacitance (RC) circuits, comprising resistive symplastic and intercellular elements and capacitive membrane elements (Grimnes and Martinsen 2015). Lossy dielectrics have a complex relative permittivity: *ε*_r_* = *ε*_r_ˊ– i × *ε*_r_˝, where *ε*_r_ˊ is the real part (energy storage by polarization) and *ε*_r_˝ is the imaginary part (energy dissipation by ionic conduction) of permittivity and i is the imaginary unit. Thus, the complex capacitance is: C* = *ε*_0_ × (*ε*_r_ˊ– i × *ε*_r_˝) × A × d^–1^. The dissipation factor (D) is the ratio of dielectric losses to energy storage: D = *ε*_r_˝/*ε*_r_ˊ = G/(*ω* × C), where G is the electrical conductance (= 1/R) and *ω* is the angular frequency. D is complementary to the phase angle (*Φ*) of impedance: D = tan(90°– *Φ*).

Root electrical capacitance (C_R_), measured between a ground electrode inserted into the growing medium, and a plant electrode fixed on the shoot base, was found to be linearly correlated with root system size in the case of the same species, substrate conditions and electrode locations (Chloupek et al. 2006; Středa et al. 2020). A few studies questioned whether dielectric properties were predictive for the whole root system due to the substantial current leakage at the proximal parts of the root–soil interface (Dietrich et al. 2012; Peruzzo et al. 2020). In contrast, others showed that the current could penetrate deep into the roots, so that most of the root system contributed to the impedance measured (Ozier-Lafontaine and Bajazet 2005; Ellis et al. 2013; Gu et al. 2021).

It is generally accepted that the electrical response depends not only on the size of the root system, but also on histological features (*e.g*. tissue density, water content, cell wall composition, lignification) related to the physiological status (Dalton 1995; Ellis et al. 2013; Peruzzo et al. 2020), indicating that C_R_ can serve as an indicator of root functional intensity (Ellis et al. 2013; Ehosioke et al. 2020; Cseresnyés et al. 2024). Stress modifies membrane composition, and increases membrane permeability and thus the conductive dielectric loss (D_R_) in roots (Li et al. 2017; Jócsák et al. 2019). This appears as an increase in root electrical conductance (G_R_), which, as AC flows in the root–substrate continuum by ionic movement, is linked to root hydraulic conductance, L_R_ (Weigand and Kemna 2019). For this reason, the dielectric characterization of plant tissues and organs has the potential to detect stress-related changes at the cellular level (Liu et al. 2021). Spectral (from Hz to MHz) impedance measurements were used to evaluate Cd toxicity in detached root segments of pea (Jócsák et al. 2010) and *Cotinus* (Xiang et al. 2018) seedlings. Only a few studies have used intact root systems for monitoring the effect of heavy metal, alkalinity or drought, but they all applied single-time measurements repeated once or twice a week on the same plants during their growth cycle (Vamerali et al. 2009; Cseresnyés et al. 2018, 2019). It is known, however, that Cd and other stressors induce faster changes in membrane surface charge density and electrical potential (Pavlovkin et al. 2006), and also in root apparent conductivity and polarization signatures (Weigand and Kemna 2019).

In this study, a pot experiment involving various species was undertaken to evaluate the usefulness of single-frequency (1 kHz) dielectric (C_R_, D_R_ and G_R_) measurements in intact root–substrate systems in a minute-scale time resolution for monitoring the short-term effect of severe Cd toxicity. This time-series methodology necessitated the design and assembly of a specialized electrical measurement system. Non-destructive shoot investigations were also performed parallel to the electrical monitoring to follow the treatment efficiency via assessing the Cd-induced changes in photosynthetic and transpiration activities, and to demonstrate the relationship between the ongoing shoot physiological disruption and root dielectric response. The general aim was to present a novel *in situ* approach for tracking stress effects through the root system.

## Materials and methods

### Plant cultivation and treatment

Maize (*Zea mays* L., cv. Mv Tarján), cucumber (*Cucumis sativus* L., cv. Perez-F1) and pea (*Pisum sativum* L., cv. Rhein dwarf) were used for the experiment. These plants are easily cultured in pots, and were reported to be convenient for toxicity tests, representing different sensitivity and response mechanisms to heavy metals (Moreno-Caselles et al. 2000, Rahoui et al. 2010, Akhtar et al. 2017). The plants were grown in 2.9 L plastic pots containing 1800 g of vermiculite–rhyolite 1.5:1 v/v mixture with a pH of 7.82, cation exchange capacity of 7.81 mmol 100 g^–1^ and 0.33 cm^3^ cm^–3^ water content at field capacity. The seeds were germinated on moistened paper towels in Petri dishes at 20 °C for 3 days in darkness, and were then planted one per pot to a depth of 1.5 cm. The plants were cultivated in a temperature- and light-controlled, 2.5 × 2.2 m growth chamber at 25/17 °C and 16/8 h light/dark cycles (light from 2 a.m. to 6 p.m.), and 60 ± 10% air humidity. Artificial illumination was provided by six metal halide lamps (Philips Master HPI-T Plus; Philips Lighting B.V., Eindhoven, The Netherlands) at about 600 μmol m^2^ s^–1^ (400–700 nm). The substrate was watered daily on a weight basis (± 1 g) to 80% of field capacity, and was fertilized twice a week with 150 mL of Hoagland’s solution. As the time-series dielectric measurement was only feasible for one plant at a time, a single seedling was cultivated per week to obtain a 28-day-old plant each week for the 7-day monitoring.

Altogether nine plants of each species were grown: three replicates for the three Cd treatments, namely control (*Cd0*), 20 mg Cd (*Cd20*) and 50 mg Cd (*Cd50*) kg^–1^ substrate. These high Cd levels were selected based on the preliminary tests (covered the range from 5 to 100 mg Cd kg^–1^) to provoke strong plant stress responses within the time frame of the dielectric measurement. Twenty-four hours before starting the electrical measurements, the substrate was irrigated with 250 mL of water (*Cd0*) or CdSO_4_ solution (*Cd20* and *Cd50*).

### Electrical measurements and plant harvest

**Fig. 1:**
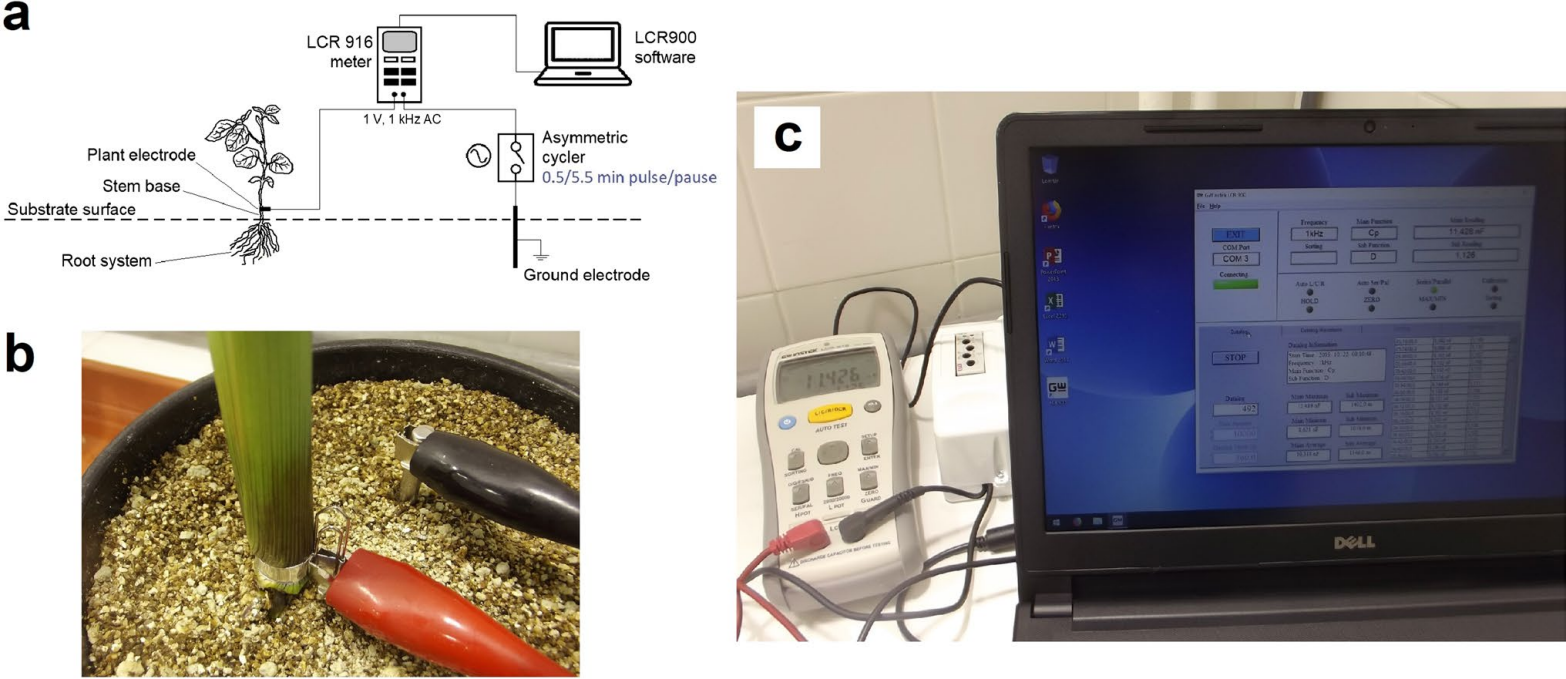
(**a**) Schematic representation of monitoring dielectric response of intact root systems on a fine timescale. (**b**) Ground and plant electrodes during the measurement process. (**c**) GW-Instek LCR-916 meter, CRM-2H asymmetric cycler and LCR900 v.1.201 data logging software

As impedance parameters are very sensitive to substrate water content (SWC), the pot was placed in a tray filled with water at a steady depth of 6 mm right after the Cd treatment to adjust and maintain SWC constant during the entire measurement period. The water content in the bulk substrate (0–12 cm; the depth of the ground electrode) was 0.26 ± 0.01 cm^3^ cm^–3^, which was checked daily using a HS2 TDR meter attached to a CS659 probe (Campbell Inc, Logan, UT, USA). This procedure allowed the substrate surface to dry to 0.12 ± 0.01 cm^3^ cm^–3^ at 0–1 cm (checked with an MO750 meter; Extech Co. Ltd., Nashua, NH, USA) to minimize AC leakage (Gu et al. 2021). The root dielectric properties (modelled as a parallel RC circuit) were monitored at 1 kHz AC with 1 V terminal voltage, using a portable GW-Instek LCR-916 meter (Goodwill Co. Ltd., Taiwan) inside the growth chamber (Fig. 1a). The ground electrode was a stainless steel rod, 15 cm long and 6 mm i.d., inserted vertically into the growing medium to 12 cm depth, at a distance of 5 cm from the plant stem (Fig. 1b). The plant electrode was clamped 1 cm above the substrate through a 4 mm wide, 25 μm thick alumina strip that bent the stem. Although the low current density formed on root membranes by the AC signal does not damage plant functions (Jócsák et al. 2019), an asymmetric cycler (CRM-2H; ETI d.d., Izlake, Slovenia) was installed between the LCR terminal and the ground electrode (Cseresnyés et al. 2024). The cycler was set to a 0.5/5.5 min pulse/pause interval to interrupt the current flow generated continuously by the LCR instrument during operation. The impedance meter was connected to a laptop supplied with LCR900 v.1.201 data logging software (Fig. 1c). Each electrical monitoring started 24 h after the Cd treatment (this time was needed to stabilize SWC), in the middle of a light period (at 10 a.m.), and lasted for 144 h (until the 168th hour). Ten C_R_ and D_R_ data per hour (one during each pulse interval of the cycler) were recorded instrumentally throughout the time series. Five consecutive data (covering 30 min) were averaged to mitigate fluctuations. A G_R_ value was calculated from each of the 288 data pairs, as C_R_ × D_R_ × *ω*.

After measurement the shoot was cut at the substrate surface. The roots were gently washed with running water and flotated to retain most of the fine roots, and were then thoroughly rinsed with distilled water three times to remove surface-bound ions. Shoot dry mass (SDM) and root dry mass (RDM) were determined after drying at 70 °C to constant weight, and root/shoot ratio (RSR) was calculated.

### Elemental analysis

The dried shoots and roots were ground to a powder. Total-N concentration was measured by Kjeldahl’s method. The concentrations of Cd, P, K, Ca, Cu, Mg, Fe and Zn were analyzed with ICP-OES (iCAP™ 7400; Thermo Fisher Sci., Cambridge, UK).

### Physiological investigations and root scanning

In order to avoid moving and touching the plants during the electrical measurements, another set of 27 plants (3 replicates × species × Cd treatment) were grown simultaneously for 28 days. The leaf Chl content, the maximum quantum efficiency of photosystem II (F_v_/F_m_) and g_s_ were measured to evaluate plant physiological status. These are considered as sensitive parameters to assess the susceptibility of plants to stress, and can be easily monitored using handheld devices (Yaniccari et al. 2012). The leaf physiological measurements were taken *in situ* in the growth chamber during the mid-light hours (from 8 a.m. to 11 a.m.), right before (day 0 = *D0*), and 2, 4 and 7 days (*D2*, *D4*, *D7*) after Cd treatment. The measurements were performed on leaves 2–5 (numbered from the oldest to the youngest) for maize, 3–7 for cucumber and 2–6 for pea, avoiding leaf edges and major veins. The total Chl content (μmol m^–2^) was recorded as the mean of three readings on the upper (adaxial) side of the leaves, using an MC-100 instrument (Apogee Inc., Logan, UT, USA). F_v_/F_m_ was determined with an OS-30p^+^ fluorometer (Opti-Sciences Inc., Hudson, NH, USA), by exposing the leaves to a saturation pulse of 6000 μmol m^2^ s^–1^ after 30 min of dark adaptation with black clips. The g_s_ value (mmol m^–2^ s^–1^) was measured on the lower (abaxial) leaf surface with an SC-1 steady state diffusion porometer (Decagon Inc., Pullmann, WA, USA).

After the last set of measurements the shoots were cut and dried for SDM. The root systems were washed, stained with methylene blue solution for 24 h and rinsed with water. The total root length (RL) was determined with the modified line intersect method (Oliveira et al. 2000). Each root system was cut into 6–15 smaller subsamples (depending on root size). The subsample was placed in a thin layer of water in a rectangular glass tray over a regular grid of 1.0 cm squares. The roots were spread to minimize branch crossings and overlaps. The total number of intersections between the roots and the horizontal and vertical gridlines was counted, and root length was calculated as: root length (cm) = π/4 × number of intersects × grid unit (cm). The length of subsamples were summed up to obtain RL. Thereafter, RDM and RSR was determined.

### Data analysis

The additive seasonal part was extracted from the time series of C_R_, D_R_ and G_R_ with STL seasonal trend decomposition based on locally estimated scatterplot smoothing, LOESS (Cleveland et al. 1990). Periodogram methods for spectral decomposition were applied in time series analysis (Shumway and Stoffer 2006). The 24-h seasonality was rejected if the s24 index was < 2, and accepted if s24 was ≥ 2. When seasonality was accepted, it was sufficient to check the trend component to test for monotonicity. When seasonality was rejected, a series of nonlinear kernel smoothing were performed with up to 24-h bandwidth, and monotonicity was only accepted if a monotonic curve was obtained after smoothing. Statistics were done using the “stl” function of R 4.0.5 (R Core Team 2021), the “periodogram” function of the “descomponer” package and the “sm.monotonicity” function of the “sm” package. The effect of Cd treatments on C_R_, D_R_ and G_R_ was analyzed by performing separate one-way ANOVA with Tukey’s post hoc test for data from each of the 288 time points. Normality, and the equality of variances in the data groups were examined with the Shapiro–Wilk test and Bartlett test, respectively.

One-way ANOVA with Tukey’s test was performed to evaluate the effect of Cd exposure on SDM, RDM, RL, RSR, and shoot and root element concentration. The nonparametric Kruskal–Wallis test with Dunn’s test was applied when the SD’s were significantly different. A repeated measures ANOVA with Tukey’s test was used to analyze the effect of Cd on Chl content, F_v_/F_m_ and g_s_ measured on various leaves. Statistical significance was assessed at *p* < 0.05 in each case.

## Results

### Time series of the dielectric properties

**Fig. 2:**
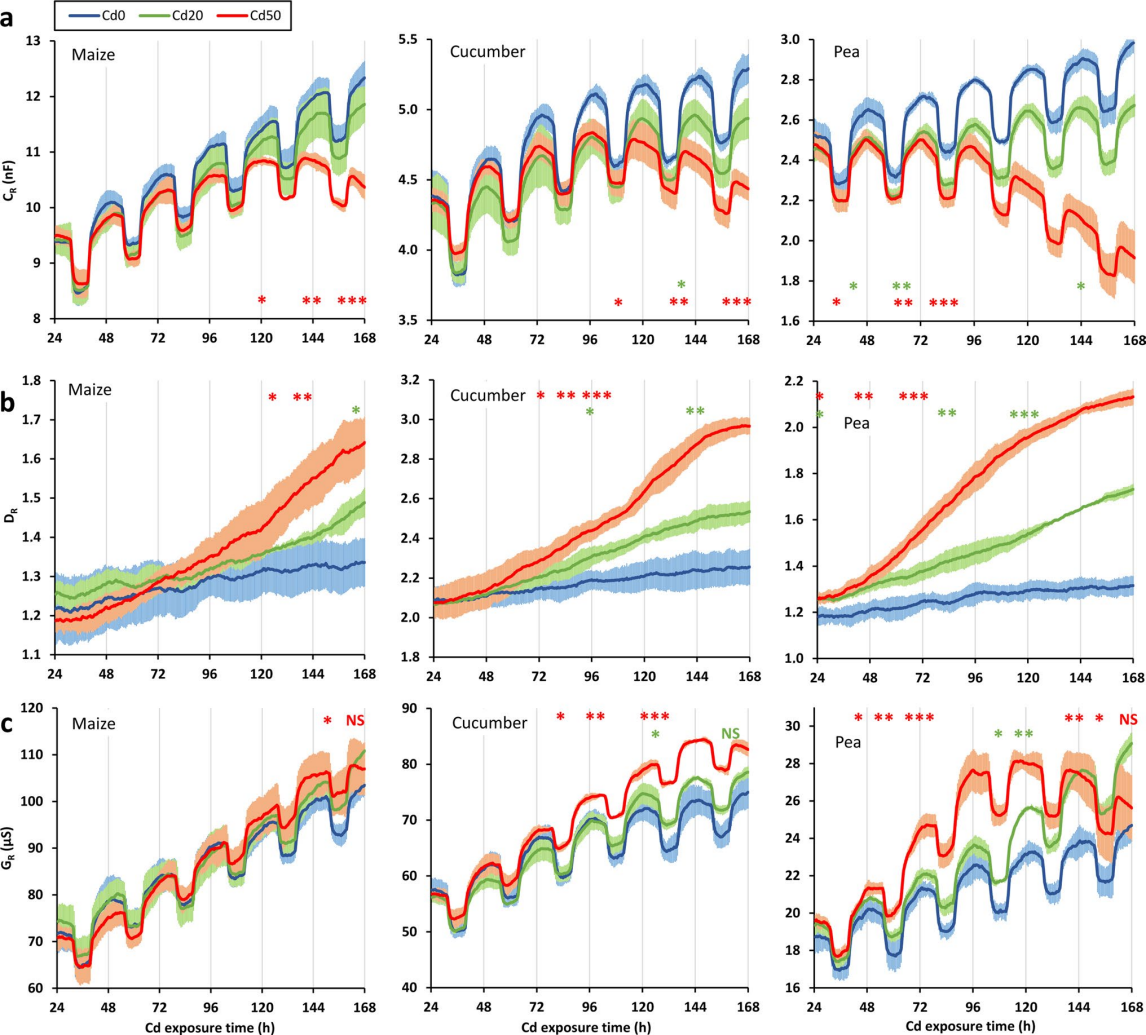
Time series of (**a**) root electrical capacitance (C_R_), (**b**) dissipation factor (D_R_) and (**c**) electrical conductance (G_R_) for maize, cucumber and pea exposed to different Cd levels (mean ± SD; n = 3). *Cd0*, *Cd20* and *Cd50*: 0 (control), 20 and 50 mg Cd kg^–1^ substrate, respectively. Asterisks (green for *Cd20* and red for *Cd50*) indicate the time point from which the Cd effect is significant at a given level compared to the control (obtained using one-way ANOVA with Tukey’s post hoc test). *p < 0.05, **p < 0.01, ***p < 0.001, NS non-significant again

The periodogram method revealed a 24-h seasonality (s24: 3.87–8.26) in the data series of C_R_ for each species and treatment, with a rapid increase and decrease at the beginning of each light and dark period, respectively (Fig. 2a, Table 1). According to the STL seasonal trend decomposition, the control (*Cd0*) plants exhibited a monotonous increasing trend in C_R_ over time, up to 131%, 121% and 118% of the initial (24.5 h) C_R_ value by the end of the measurement period (168 h) in the case of maize, cucumber and pea, respectively. The *Cd20* plants also showed a monotonous increase in C_R_, but with a slower rate; the last C_R_ values were 126%, 114% and 109% of those first measured for the above species. The trend, however, was found to be non-monotonic for the *Cd50* treatments of all the species. In maize and cucumber, C_R_ progressively increased until reaching a maximum at 141 h and 97 h, respectively, and then decreased to 109% and 102% of the initial value by the end of the measurement. In pea, there was no increasing trend in C_R_ at all; almost the same peaks were measured during the first three mid-light periods around 24, 48 and 72 h, followed by a decrease to a lower level at 168 h (77% of the first C_R_ value recorded). Compared to the *Cd0* control, the *Cd20* treatment had no significant effect on C_R_ at any of the measurement time points for maize, but significantly reduced C_R_ from 139 h onwards for cucumber, and much earlier, from 44 h for pea until the end of the monitoring period. The *Cd50* treatment significantly decreased C_R_ permanently from 121 h, 113 h and as early as 36.5 h for maize, cucumber and pea plants, respectively.

**Table 1:** Statistical results for the time series of root electrical capacitance (C_R_), dissipation factor (D_R_) and electrical conductance (G_R_) for maize, cucumber and pea exposed to different Cd levels (n = 3). *Cd0*, *Cd20* and *Cd50*: 0 (control), 20 and 50 mg Cd kg^–1^ substrate, respectively. STL seasonal trend decomposition was used to extract the additive seasonal part from the time series. The s24 index (s24) was calculated using the periodogram method. If the 24-h seasonality was accepted (s24 ≥ 2; written in bold), the trend component was simply checked for monotonicity. Otherwise, a series of nonlinear kernel smoothing with up to 24-h bandwidth was used for monotonicity

Parameter	Cd level	Maize		Cucumber		Pea	
		s24	Trend	s24	Trend	s24	Trend
C_R_	Cd0	**4.80**	Increasing	**6.66**	Increasing	**5.61**	Increasing
	Cd20	**4.62**	Increasing	**8.01**	Increasing	**8.26**	Increasing
	Cd50	**3.87**	Not monotonic	**5.40**	Not monotonic	**4.97**	Not monotonic
D_R_	Cd0	**2.83**	Increasing	0.68	Increasing	**2.61**	Increasing
	Cd20	0.65	Increasing	0.24	Increasing	0.06	Increasing
	Cd50	0.07	Increasing	0.01	Increasing	0.19	Increasing
G_R_	Cd0	**5.90**	Increasing	**7.26**	Increasing	**6.43**	Increasing
	Cd20	**5.02**	Increasing	**8.84**	Increasing	**8.34**	Increasing
	Cd50	**3.82**	Increasing	**5.92**	Increasing	**5.33**	Not monotonic

The time series of D_R_ showed significant 24-h seasonality only for control maize (s24: 2.83) and pea (s24: 2.61), with small local maxima during the mid-light periods (Fig. 2b, Table 1). A monotonous increasing trend in D_R_ over time was found for each species and treatment. Depending on dosage, the addition of Cd provoked an increase in D_R_. The difference in D_R_ from *Cd0* controls became statistically significant from 165 h (*Cd20*) and 126.5 h (*Cd50*) for maize, from 96 h (*Cd20*) and 74 h (*Cd50*) for cucumber, and right from the beginning of the dielectric measurement (24.5 h) for both Cd treatments on pea.

Each data series of G_R_ showed significant 24-h seasonality (s24: 3.82–8.84; Fig. 2c, Table 1), which evidently corresponded with the light/dark variations of C_R_. The trend in G_R_ was always monotonously increasing, except for the *Cd50* treatment on pea. In the latter case there was no monotonic trend in the series: after a sharp increase, G_R_ reached a plateau around 117 h, and then began to drop markedly. Compared to the controls, the *Cd20* treatment did not significantly influence G_R_ at any time point for maize, but increased it transitionally (from 129 to 160 h) for cucumber, and from 105.5 h until the end of the measurements for pea. The *Cd50* treatment only resulted in a significant increase in G_R_ for a short period of time (from 153.5 to 160 h) for maize, but permanently from 81.5 h for cucumber, and from 45.5 to 156 h for pea plants.

### Shoot symptoms and physiological response

Obvious toxicity symptoms appeared on the aboveground plant parts by the end of the 7-day Cd exposure. Maize leaves began to curl, and turned yellow, chiefly on the tips and edges. The lower leaves of cucumber were slightly wilted, and became chlorotic around the main veins. The most severe damage was visible in pea: each leaf and the shoot tip wilted, and the two or three oldest leaves dried up.

**Fig. 3:**
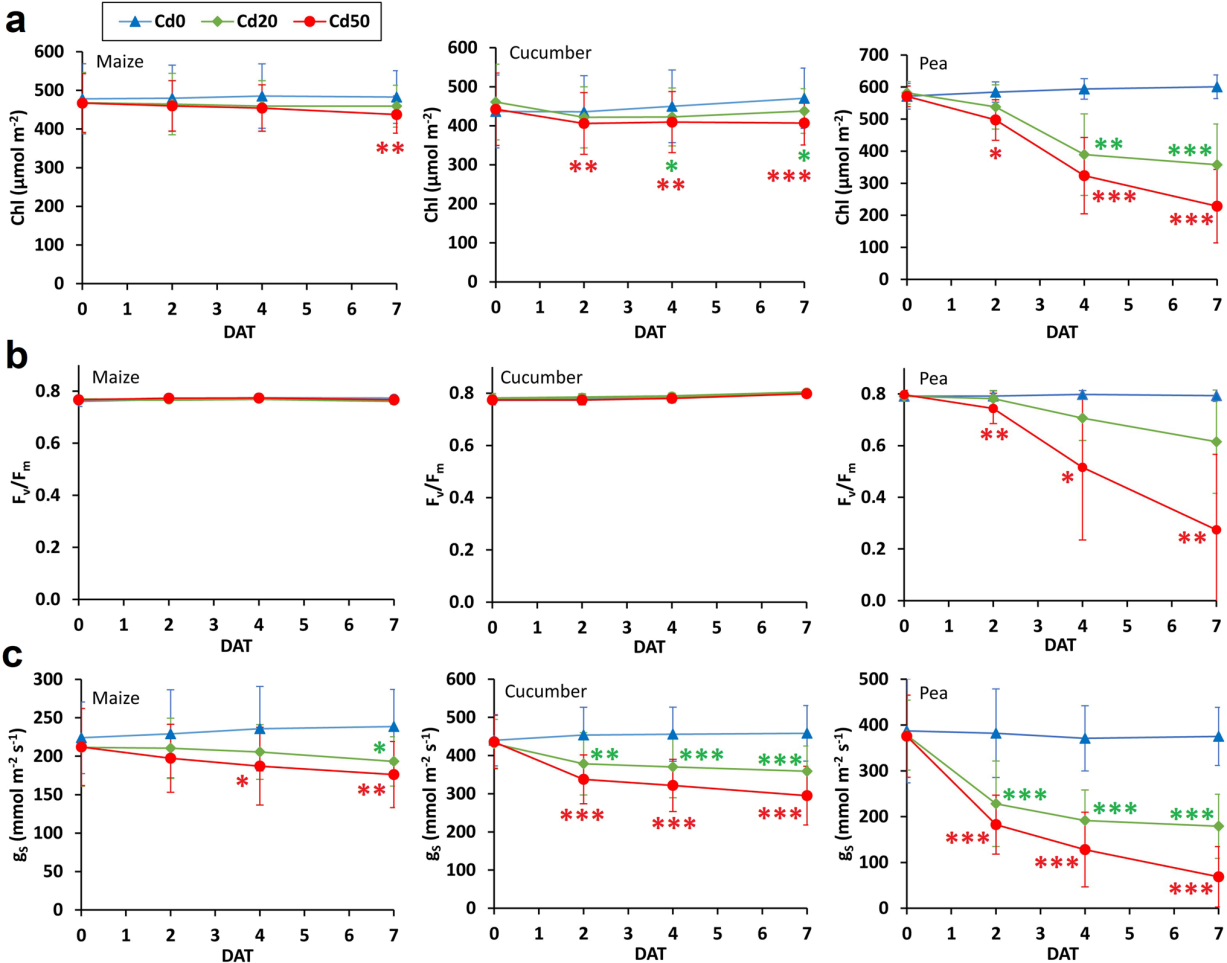
(**a**) Changes in the total chlorophyll (Chl) content, (**b**) maximum quantum efficiency of photosystem II (F_v_/F_m_) and (**c**) stomatal conductance (g_s_) over time (DAT: days after treatment) in maize, cucumber and pea plants exposed to different Cd levels. *Cd0*, *Cd20* and *Cd50*: 0 (control), 20 and 50 mg Cd kg^–1^ substrate, respectively. The values were averaged across leaves and replicate plants (mean ± SD; n = 3) for clarity. Asterisks (green for *Cd20* and red for *Cd50*) represent significant differences compared to the control (obtained using repeated-measures ANOVA with Tukey’s test). *p < 0.05, **p < 0.01, ***p < 0.001

Cd toxicity was manifested as reduced leaf Chl content, F_v_/F_m_ and g_s_, particularly in pea (Fig. 3). The Chl concentration in maize leaves only decreased significantly at the higher Cd level at *D7*, compared to the controls. A quicker, stronger response to Cd was observed in cucumber and pea: the Chl content significantly decreased from *D4* and from *D2* in the *Cd20* and *Cd50* treatments, respectively. The stress-induced reduction in F_v_/F_m_ proved to be statistically significant only for the *Cd50* treatment on pea, but from *D2* onwards in this case. Cd exposure had a marked negative effect on g_s_: the decrease under stress was significant at *D7* for *Cd20* and at *D4* and *D7* for *Cd50* maize plants, compared to the *Cd0* controls, but both Cd levels resulted in a significant impact already from *D2* onwards in cucumber and pea.

### Shoot and root biomass and total root length

**Fig. 4:**
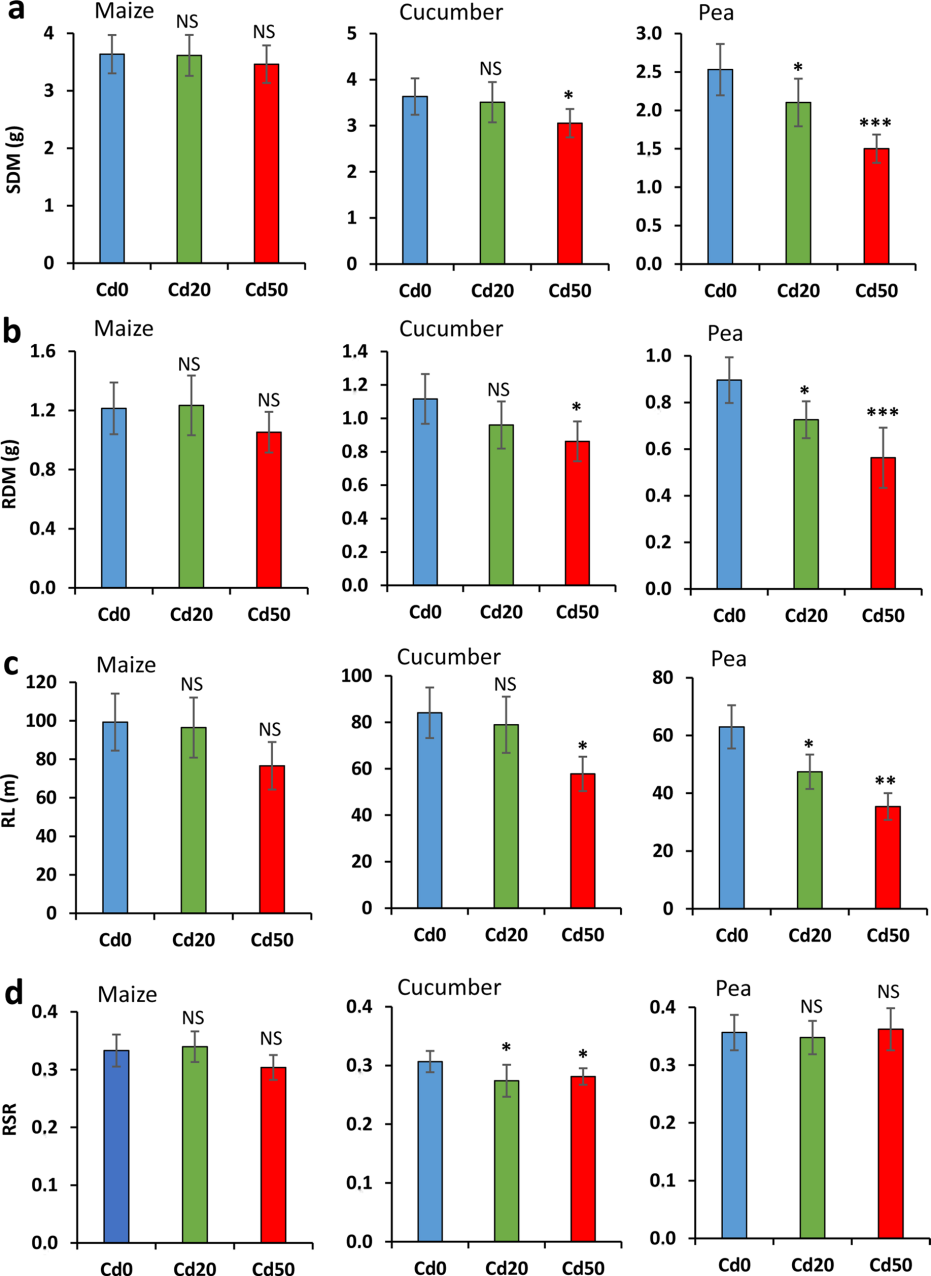
(**a**) Shoot dry mass (SDM; mean ± SD; n = 6), (**b**) root dry mass (RDM; n = 6), (**c**) total root length (RL; n = 3) and (**d**) root/shoot ratio (RSR; n = 6) for maize, cucumber and pea exposed to different Cd levels for 7 days. *Cd0*, *Cd20* and *Cd50*: 0 (control), 20 and 50 mg Cd kg^–1^ substrate, respectively. Asterisks represent significant differences from the controls obtained using one-way ANOVA with Tukey’s test. *p < 0.05, **p < 0.01, ***p < 0.001, NS non-significant

Cd addition had no significant influence on the SDM, RDM, RL or RSR of maize (Fig. 4). However, the *Cd50* treatment led to a non-significant, 13% and 23% decrease in RDM and RL, respectively, compared to the *Cd0* controls. In cucumber, the effect of the low Cd level on shoot and root growth proved to be statistically insignificant, whereas the high Cd dose resulted in a significant, 16%, 23% and 31% decrease in SDM, RDM and RL, respectively. RSR significantly reduced by both Cd treatments. Among the species, the biomass production of pea was the most affected by Cd stress: compared to the control plants, the *Cd20* treatment significantly reduced SDM by 17%, RDM by 19% and RL by 25%, while the corresponding changes were 41%, 37% and 44% in the case of the *Cd50* treatment. No significant changes in RSR by Cd was found.

### Shoot and root element concentration

**Fig. 5:**
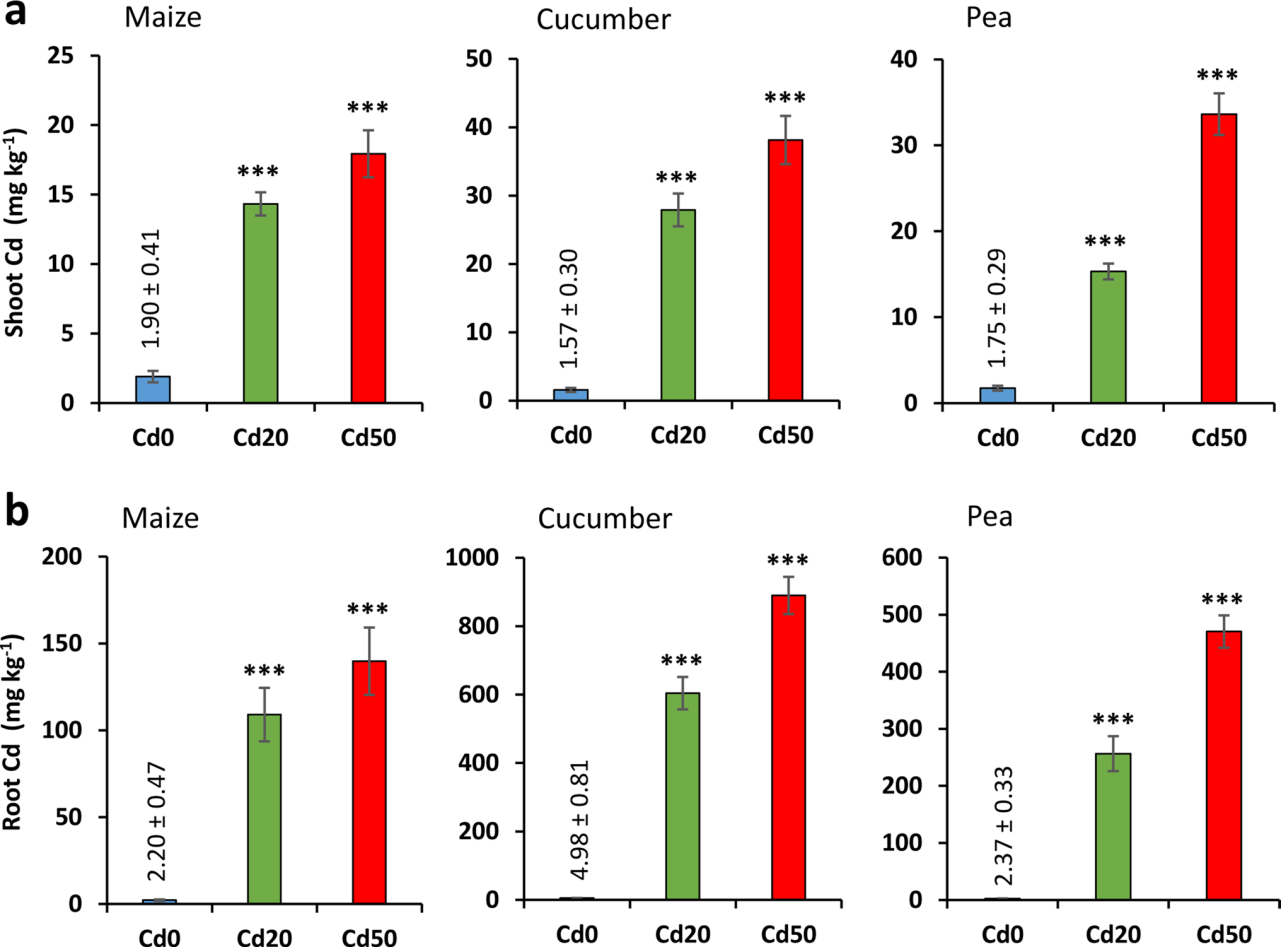
Cd concentrations (mean ± SD; n = 3) in dry (**a**) shoot and (**b**) root samples of maize, cucumber and pea exposed to different Cd levels for 7 days. *Cd0*, *Cd20* and *Cd50*: 0 (control), 20 and 50 mg Cd kg^–1^ substrate, respectively. ***: significantly different from the controls at the* p* < 0.001 level, obtained using the nonparametric Kruskal–Wallis test with Dunn’s test

ICP-OES analysis demonstrated a highly significant increase in the shoot and root Cd concentration at increasingly high Cd treatment levels (Fig. 5). The Cd concentration in dry shoot biomass reached 17.9, 38.1 and 33.6 mg kg^–1^ in the *Cd50* treatments of maize, cucumber and pea, respectively, compared to the 1.6–1.9 mg kg^–1^ values measured in the control plants. A substantially higher amount of Cd was accumulated in the roots: 140, 889 and 470 mg kg^–1^ Cd was determined in *Cd50* maize, cucumber and pea, respectively, compared to 2.2–5.0 mg kg^–1^ Cd found in the *Cd0* controls. Both the Cd doses significantly decreased the concentration of all the investigated elements except P in pea root, and reduced the N, P, K, Cu and Zn in the shoot (Table S1). In cucumber the effect was only significant for Ca, Cu, Fe and Zn, whereas the element concentration of maize root was not affected statistically by the Cd treatments (it should be noted that the low replicate number may have limited the level of significance in many cases).

## Discussion

### Root dielectric response to Cd

Cell membrane capacitors were reported to show constant or slightly decreasing C with rising temperature due to increasing polarization loss (Grimnes and Martinsen 2015). Therefore, the marked 24-h seasonality in the C_R_ data series was attributed to the cyclic root water uptake activity strongly linked to the light/dark changes in canopy transpiration (Henzler et al. 1999), as was previously verified by modifying temperature regime and light cycles under chamber conditions (Cseresnyés et al. 2024). Diurnal variation in plant hydraulics is driven by endogenous circadian regulation coincidently with external temperature and photoperiod cues (Greenham and McClung 2015). The present experimental results supported the previous hypothesis that C_R_ is influenced by both the root functional status and the geometrical size (Dalton 1995; Ehosioke et al. 2020). The increasing trend in C_R_ indicated an extension of the root system, especially for maize maintaining intensive vegetative growth. Depending on the level applied, Cd significantly reduced C_R_ compared to the control plants, and interrupted the increasing trend in the time series. Cd excess in the rhizosphere impedes root cell division and root-hair production, and inhibits root initiation and elongation, leading to the formation of shorter, thicker root branches (Lux et al. 2011). Accelerated exo- and endodermal maturation and the deposition of lignin and suberin closer to the root apices, together with an increase in epidermal and cortical thickness provide a more efficient barrier to Cd transport into the vascular cylinder and thus to the shoot (Hose et al. 2001; Díaz et al. 2021). Basically, there are three reasons for the Cd-induced reduction in the magnitude of C_R_. First, Cd decreased RL and the root surface area, *i.e*. the area of the polarizable membrane dielectrics (A) in the capacitor. Second, lignin and suberin have much smaller *ε*_r_ (from 2 to 2.4) than the other main root constituents, in particular cellulose (*ε*_r_ ~ 7.6) and water (*ε*_r_ ~ 80) (Ellis et al. 2013). Enhanced suberization, and the lower root water content, which is another typical consequence of Cd toxicity (Xiang et al. 2018) or soil alkalinity (Cseresnyés et al. 2019), decrease the resultant *ε*_r_ of root tissue. Third, restricted root elongation reduced the ratio of young, absorptive root parts to older, more suberized transporting segments, leading to a lower integrated water uptake rate in the stressed root system (Lobet et al. 2013).

In some cases, D_R_ values increased over time up to the mid-light hours. Depending on histological features and environmental conditions, water flows across the root cylinder through a variable combination of hydrostatically driven apoplastic, and mainly osmotically controlled (aquaporin-mediated) cell-to-cell (*i.e*. symplastic and transmembrane) pathways (Sivasakthi et al. 2020). During daylight the higher transpiration pull and xylem tension increase the ratio of the primarily electrically resistive apoplastic flow to the more capacitive cell-to-cell movement (Lobet et al. 2013), leading to enhanced conductive energy loss, D_R_. However, the present results for cucumber question the efficiency of using dielectric measurements to reveal clear diurnal cycles in D_R_ under the experimental conditions applied here. In Cd-treated plants, enhanced leaf senescence and reduced light transpiration (detected via g_s_) was thought to be responsible for the cessation of seasonality in the D_R_ time series. Although tissue development during root ageing always causes some increase in D_R_ detected at 1 kHz AC (Ehosioke et al. 2023), more intensive changes were observed when plants were exposed to various substrate Cd levels (Cseresnyés et al. 2018). Cd toxicity triggers rapid membrane depolarization in the outer cortical cells, and induces membrane lipid peroxidation due to excessive ROS production, altering membrane structure and fluidity (Artiushenko et al. 2014). Depolarization by the Cd levels applied was accompanied by a marked increase in membrane hydraulic conductivity and by substantial electrolyte leakage from the cells even 24–48 h after treatment in maize (Pavlovkin et al. 2006), cucumber (Kabała et al. 2008) and pea roots (Lehotai et al. 2011), as was demonstrated in the present study by significantly higher D_R_ values. Enhanced membrane permeability, the death of root cortical cells and the resulting H_2_O_2_-induced aerenchyma formation caused by Cd toxicity was reported to modify the impedance response, manifested as reduced intracellular resistance (Xiang et al. 2018) or *Φ* (Ehosioke et al. 2023).

The light/dark pattern in the G_R_ time series is likely linked to the fluctuation of L_R_ (Weigand and Kemna 2019), which is coincident with the diurnal oscillation of aquaporin abundance and also of the stomatal closure and transpiration rate (Henzler et al. 1999). Notably, increasing temperature, per se, increases electrolyte G, but light/dark cycles in G_R_ have also been observed previously under constant temperature (yet unpublished results). Although the restricted root development caused by Cd treatment was over-compensated by the enhanced electrical conductivity of the disrupted membranes (Rahoui et al. 2010), leading to a transient increase in G_R_, the measured values began to decrease thereafter due to accelerated senescence and the loss of transpiration, principally for pea.

### Physiological and growth responses in relation to Cd tolerance

Besides the impedance response detected, the visible toxicity symptoms on the shoot, the leaf physiological traits, and the SDM, RDM and RL measurements clearly indicated the serious adverse effect of the two Cd doses applied. Previous studies on maize (Akhtar et al. 2017), cucumber (Moreno-Caselles et al. 2000) and pea (Hattab et al. 2009) reported that an increase in the Cd dose added to the medium of developing plants provoked a gradual decline in these parameters, and that the effect became more pronounced with the exposition time. Furthermore, in accordance with the present findings, g_s_ showed a more rapid and prominent response to Cd stress, followed by a reduction in Chl concentration and later in F_v_/F_m_ (Wang et al. 2009; Chaneva et al. 2010). The quick stomatal closure and transpiration loss, and the consequent reduced water uptake rate were convincingly demonstrated by the C_R_ and G_R_ data series. A much higher amount of Cd was found in the roots than in the shoots. This was due to the binding and retention of Cd^2+^ by negatively charged, strongly suberized cell walls, and also to the chelation and sequestration of metal ions into the vacuoles (Lux et al. 2011). Plants evolved these defense mechanisms to restrict the radial movement of Cd to xylem vessels to protect shoots from Cd loads (Hose et al. 2001).

Root dielectric responses clearly indicated differences in Cd sensitivity between the studied species. The C_R_ and G_R_ time series themselves showed that pea was the most rapidly and seriously impacted by Cd excess: the stressed plants exhibited the highest relative decrease in C_R_ compared to the controls by the end of treatment (Table S2), with a significant treatment effect for both metal levels. Supporting this finding, the degree of visible shoot damage, and the percentage changes in physiological and biomass parameters were the greatest for pea among the three species. Being an important food crop, pea is widely used in pollution tests due to its high sensitivity to Cd (Hattab et al. 2009; Rahoui et al. 2010). It has been previously reported for pea that a week of Cd exposure resulted in substantial endodermal suberin deposition and lamellae formation, a reduced number and length of lateral roots, and characteristic root browning (Rodríguez-Serrano et al. 2006; Głowacka et al. 2019). These results concur with the present dielectric monitoring, and with post-harvest root measurements and visual observations. Although root architecture has not been evaluated, the higher percentage reduction in RL than in RDM implied that Cd inhibited lateral root initiation and elongation.

Cucumber seemed less susceptible to stress: the Cd loads were observed to have somewhat smaller impacts on dielectric, physiological and growth responses, with significant differences mainly in the *Cd50* treatment vs. controls. However, fast stomatal closure was detected for the *Cd20* treatment as well, in agreement with earlier literature (Sun et al. 2015). Maize proved to be the most Cd-tolerant plant on the basis of the latest stress response and the lowest relative change both in C_R_ and in the other measured properties. Maize is, in fact, known to be a promising Cd-accumulator crop with the ability to tolerate up to 45 mg Cd per kg root dry weight without exhibiting visible injury symptoms, although the tolerance level to Cd and other soil-borne stresses varies greatly among genotypes (Akhtar et al. 2017; Klimešová et al. 2020).

The macro- and micronutrient analysis of shoot and root samples confirmed the different Cd sensitivity levels of the plant species tested. Cd excess triggers a rapid K^+^ efflux from the cells through damaged membranes (Pavlovkin et al. 2006), and was found to displace Ca^2+^ from the cell walls (Lehotai et al. 2011). By competing for the same plasma membrane transporters, Cd reduces the uptake and transport of many elements, including N, P, K, Ca, Cu, Fe, Mg, Mn and Zn, leading to serious nutrient deficiency and imbalance in plants (Rizwan et al. 2016). In the present case, shoot and root element composition was influenced by Cd excess to the greatest and least extent in pea (sensitive) and maize (tolerant), respectively (Table S1).

### Conclusions and prospects

Low-frequency (1 kHz) dielectric monitoring with fine time resolution was performed over a 6-day period in intact root–substrate systems, using a specialized two-terminal measurement set-up on different plants under chamber conditions. The experimental results supported the hypothesis that this novel impedance measurement technique was suitable for tracking real-time changes, including diurnal patterns in root water uptake activity *in situ*. Moreover, this non-invasive method proved to be a reliable diagnostic tool to monitor the short-term response of plants to stress targeting the roots, such as the addition of Cd to the growing medium. The different susceptibility of the studied species to various substrate Cd levels could also be evaluated dynamically. The single-frequency dielectric monitoring is possible using a cheap handheld LCR device. Nevertheless, maintenance of stable SWC with partially dried surface (to avoid current leakage at the root neck) is an important limitation of the method application. Although impedance parameters per se serve as indicators of some aspects of root histological and functional changes, the dielectric approach can also be easily integrated into conventional and advanced plant physiological investigations. Not only heavy metals but other root stress factors, such as nutrient deficiencies, anomalies in soil temperature and pH, agrochemicals and various soil pollutants, can be potentially evaluated *in situ* without affecting plant life functions. From an agricultural point of view, the present method could promote the screening and selection of crop genotypes with improved stress tolerance and consequently yield potential. Furthermore, the dielectric assessment of tolerance and acclimatory response to heavy metal pollution may help in evaluating the phytoremediation potential of plants, contributing to more efficient practical use. Although single-time impedance measurements have been performed successfully for several field-grown crops, the adaptation of the continuous monitoring presented here to field conditions (*i.e*. variable soil moisture) will be a challenging task requiring much time and effort.

### Supplementary Information

Below is the link to the electronic supplementary material.

Supplementary file1 (PDF 249 KB)

## Data Availability

The data that support the findings of this study are available from the corresponding author upon reasonable request.

## Abbreviations

A: Capacitor plate area
AC: Alternating current
C: Electrical capacitance
Chl: Chlorophyll
C_R_: Electrical capacitance of root–substrate system
d: Capacitor plate separation
D: Dissipation factor
D_R_: Dissipation factor of root–substrate system
F_v_/F_m_: Maximum quantum yield of PSII photochemistry
G: Electrical conductance
G_R_: Electrical conductance of root–substrate system
g_s_: Stomatal conductance
L_R_: Root hydraulic conductance
R: Electrical resistance
RDM: Root dry mass
RL: Total root length
ROS: Reactive oxygen species
RSR: Root/shoot ratio
SDM: Shoot dry mass
SWC: Substrate water content
*ε*_0_: Vacuum permittivity
*ε*_r_: Relative permittivity
*Φ*: Impedance phase angle
*ω*: Angular frequency

## References

[CR1] Akhtar T, Zia-ur-Rehman M, Naeem A, Nawaz R, Ali S, Murtaza G, Maqsood MA, Azhar M, Khalid H, Rizwan M (2017). Photosynthesis and growth response of maize (*Zea** mays* L.) hybrids exposed to cadmium stress. Environ Sci Pollut Res.

[CR2] Artiushenko T, Syshchykov D, Gryshko V, Čiamporová M, Fiala R, Repka V, Martinka M, Pavlovkin J (2014). Metal uptake, antioxidant status and membrane potential in maize roots exposed to cadmium and nickel. Biologia.

[CR3] Bali AS, Sidhu GPS, Aftab T, Hakeem KR (2022). Cadmium uptake, toxicity, and tolerance in plants. Heavy Metal Toxicity in Plants. Physiological and Molecular Adaptations.

[CR4] Bali AS, Sidhu GPS, Kumar V (2020). Root exudates ameliorate cadmium tolerance in plants: a review. Environ Chem Lett.

[CR5] Chaneva G, Parvanova P, Tzvetkova N, Uzunova A (2010). Photosynthetic response of maize plants against cadmium and paraquat impact. Water Air Soil Poll.

[CR6] Chloupek O, Forster BP, Thomas WTB (2006). The effect of semi-dwarf genes on root system size in field-grown barley. Theor Appl Genet.

[CR7] Cleveland RB, Cleveland WS, McRae JE, Terpenning I (1990). STL: A seasonal-trend decomposition. J Offic Stat.

[CR8] Cseresnyés I, Rajkai K, Takács T, Vozáry E (2018). Electrical impedance phase angle as an indicator of plant root stress. Biosyst Eng.

[CR9] Cseresnyés I, Takács T, Sepovics B, Kovács R, Füzy A, Parádi I, Rajkai K (2019). Electrical characterization of the root system: a noninvasive approach to study plant stress responses. Acta Physiol Plant.

[CR10] Cseresnyés I, Füzy A, Kabos S, Kelemen B, Rajkai K, Takács T (2024). Monitoring of plant water uptake by measuring root dielectric properties on a fine timescale: diurnal changes and response to leaf excision. Plant Methods.

[CR11] Dalton FN (1995). In-situ root extent measurements by electrical capacitance methods. Plant Soil.

[CR12] Díaz AS, da Cunha Cruz Y, Duarte VP, de Castro EM, Magalhães PC, Pereira FJ (2021). The role of reactive oxygen species and nitric oxide in the formation of root cortical aerenchyma under cadmium contamination. Physiol Plant.

[CR13] Dietrich RC, Bengough AG, Jones HG, White PJ (2012). A new physical interpretation of plant root capacitance. J Exp Bot.

[CR14] Ehosioke S, Nguyen F, Rao S, Kremer T, Placencia-Gomez E, Huisman JA, Kemna A, Javaux M, Garré S (2020). Sensing the electrical properties of roots: A review. Vadose Zone J.

[CR15] Ehosioke S, Garré S, Huisman JA, Zimmermann E, Placencia-Gomez E, Javaux M, Nguyen F (2023). Spectroscopic approach toward unraveling the electrical signature of roots. J Geophys Res-Biogeo.

[CR16] Ellis T, Murray W, Kavalieris L (2013). Electrical capacitance of bean (*Vicia*
*faba*) root systems was related to tissue density – a test for the Dalton Model. Plant Soil.

[CR17] Głowacka K, Źróbek-Sokolnik A, Okorski A, Najdzion J (2019). The effect of cadmium on the activity of stress-related enzymes and the ultrastructure of pea roots. Plants.

[CR18] Greenham K, McClung CR (2015). Integrating circadian dynamics with physiological processes in plants. Nat Rev Genet.

[CR19] Grimnes S, Martinsen ØG (2015). Bioimpedance and Bioelectricity Basics.

[CR20] Gu H, Liu L, Butnor JR, Sun H, Zhang X, Li C, Liu X (2021). Electrical capacitance estimates crop root traits best under dry conditions – a case study in cotton (*Gossypium*
*hirsutum* L.). Plant Soil.

[CR21] Hattab S, Dridi B, Chouba L, Kheder MB, Bousetta H (2009). Photosynthesis and growth responses of pea *Pisum*
*sativum* L. under heavy metal stress. J Environ Sci.

[CR22] Henzler T, Waterhouse RN, Smyth AJ, Carvajal M, Cooke DT, Schäffner AR, Steudle E, Clarkson DT (1999). Diurnal variations in hydraulic conductivity and root pressure can be correlated with the expression of putative aquaporins in the roots of *Lotus*
*japonicus*. Planta.

[CR23] Hose E, Clarkson DT, Steudle E, Schreiber L, Hartung W (2001). The exodermis: a variable apoplasic barrier. J Exp Bot.

[CR24] Jócsák I, Droppa M, Horváth G, Bóka K, Vozáry E (2010). Cadmium- and flood-induced anoxia stress in pea roots measured by electrical impedance. Z Naturforsch C.

[CR25] Jócsák I, Végvári G, Vozáry E (2019). Electrical impedance measurement on plants: a review with some insights to other fields. Theor Exp Plant Physiol.

[CR26] Kabała K, Janicka-Russak M, Burzyński M, Kłobus G (2008). Comparison of heavy metal effect on the proton pumps of plasma membrane and tonoplast in cucumber root cells. J Plant Physiol.

[CR27] Klimešová J, Holková L, Středa T (2020) Drought stress response in maize: molecular, morphological and physiological analysis of tolerant and sensitive genotypes. Maydica 65:1264. https://journals-crea.4science.it/index.php/maydica/article/view/2024/1264. Accessed 26 Feb 2024

[CR28] Kubier A, Wilkin RT, Pichler T (2019). Cadmium in soils and groundwater: a review. Appl Geochem.

[CR29] Lehotai N, Pető A, Bajkán S, Erdei L, Tari I, Kolbert Z (2011). In vivo and in situ visualization of early physiological events induced by heavy metals in pea root meristem. Acta Physiol Plant.

[CR30] Li MQ, Li JY, Wei XH, Zhu WJ (2017). Early diagnosis and monitoring of nitrogen nutrition stress in tomato leaves using electrical impedance spectroscopy. Int J Agr Biol Eng.

[CR31] Liu Y, Li DM, Qian J, Di B, Zhang G, Ren ZH (2021). Electrical impedance spectroscopy (EIS) in plant root research: a review. Plant Methods.

[CR32] Lobet G, Hachez C, Chaumont F, Javaux M, Draye X, Eshel A, Beeckman T (2013). Root water uptake and water flow in the root–soil domain. Plant roots: The hidden half.

[CR33] Lux A, Martinka M, Vaculík M, White PJ (2011). Root responses to cadmium in the rhizosphere: a review. J Exp Bot.

[CR34] McLaughlin MJ, Smolders E, Zhao FJ, Grant C, Montalvo D (2021). Managing cadmium in agricultural systems. Adv Agron.

[CR35] Moreno-Caselles J, Moral R, Pérez-Espinosa A, Pérez-Murcia MD (2000). Cadmium accumulation and distribution in cucumber plant. J Plant Nutr.

[CR36] Oliveira MRG, van Noordwijk M, Gaze SR, Brouwer G, Bona S, Mosca G, Hairiah K, Smit AL, Bengough AG, Engels C, van Noordwijk M, Pellerin S, van de Geijn SC (2000). Auger sampling, ingrowth cores and pinboard methods. Root Methods: A Handbook.

[CR37] Ozier-Lafontaine H, Bajazet T (2005). Analysis of root growth by impedance spectroscopy (EIS). Plant Soil.

[CR38] Pavlovkin J, Luxová M, Mistríková I, Mistrík I (2006). Short- and long-term effects of cadmium on transmembrane electric potential (*E*_m_) in maize roots. Biologia.

[CR39] Peruzzo L, Chou C, Wu Y, Schmutz M, Mary B, Wagner FM, Petrov P, Newman G, Blancaflor EB, Liu X, Ma X, Hubbard S (2020). Imaging of plant current pathways for non-invasive root phenotyping using a newly developed electrical current source density approach. Plant Soil.

[CR40] R Core Team (2021) A language and environment for statistical computing. Vienna, Austria: R Foundation for Statistical Computing. http://www.R-project.org/. Accessed 5 Sept 2021

[CR41] Rahoui S, Chaoui A, El Ferjani E (2010). Membrane damage and solute leakage from germinating pea seed under cadmium stress. J Hazard Mater.

[CR42] Rizwan M, Ali S, Abbas T, Zia-ur-Rehman M, Hannan F, Keller C, Al-Wabel MI, Ok YS (2016). Cadmium minimization in wheat: A critical review. Ecotox Environ Saf.

[CR43] Rodríguez-Serrano M, Romero-Puertas MC, Zabalza A, Corpas FJ, Gómez M, del Río LA, Sandalio LM (2006). Cadmium effect on oxidative metabolism of pea (*Pisum*
*sativum* L.) roots. Imaging of reactive oxygen species and nitric oxide accumulation *in*
*vivo*. Plant Cell Environ.

[CR44] Sahoo RK, Pradhan M, Palei M, Maitra S, Aftab T, Hakeem KR (2022). Responses and adaptation of photosynthesis and respiration under heavy metal stress. Heavy Metal Toxicity in Plants. Physiological and Molecular Adaptations.

[CR45] Shumway RH, Stoffer DS (2006). Time Series Analysis and Its Applications.

[CR46] Sivasakthi K, Tharanya M, Zaman-Allah M, Kholová J, Thirunalasundari T, Vadez V (2020). Transpiration difference under high evaporative demand in chickpea (*Cicer*
*arietinum* L.) may be explained by differences in the water transport pathway in the root cylinder. Plant Biol.

[CR47] Středa T, Haberle J, Klimešová J, Klimek-Kopyra A, Středová H, Bodner G, Chloupek O (2020). Field phenotyping of plant roots by electrical capacitance – a standardized methodological protocol for application in plant breeding: a review. Int Agrophys.

[CR48] Sun S, Li M, Zuo J, Jiang W, Liu D (2015). Cadmium effects on mineral accumulation, antioxidant defense system and gas exchange in cucumber. Zemdirbyste.

[CR49] Vamerali T, Bandiera M, Coletto L, Zanetti F, Dickinson NM, Mosca G (2009). Phytoremediation trials on metal- and arsenic-contaminated pyrite wastes (Torviscosa, Italy). Environ Pollut.

[CR50] Wang H, Zhao SC, Liu RL, Zhou W, Jin JY (2009). Changes of photosynthetic activities of maize (*Zea*
*mays* L.) seedlings in response to cadmium stress. Photosynthetica.

[CR51] Weigand M, Kemna A (2019). Imaging and functional characterization of crop root systems using spectroscopic electrical impedance measurements. Plant Soil.

[CR52] Xiang D, Zhang G, Gong R, Di B, Tian Y (2018). Effect of cadmium stress on growth and electrical impedance spectroscopy parameters of *Cotinus*
*coggygria* roots. Water Air Soil Pollut.

[CR53] Yaniccari M, Tambussi E, Istilart C, Castro AM (2012). Glyphosate effects on gas exchange and chlorophyll fluorescence responses of two *Lolium*
*perenne* L. biotypes with different herbicide sensitivity. Plant Physiol Biochem.

