# Supplementary material for: Detection of plant cadmium toxicity by monitoring dielectric response of intact root systems on a fine timescale
Source: Environ Sci Pollut Res Int. 2024 Apr 12;31(21):30555–68. doi: 10.1007/s11356-024-33279-w (PMC11096224; doi:10.1007/s11356-024-33279-w)
Supplement: Supplementary file 1 — Supplementary file1 (PDF 249 KB) [file 11356_2024_33279_MOESM1_ESM.pdf]

Table S1: Chemical element concentrations (mean ± SD; n = 3) in dry shoot and root samples of maize, cucumber and pea exposed to different Cd levels for 7 days. *Cd0*, *Cd20* and *Cd50*: 0 (control), 20 and 50 mg Cd kg^{-1} substrate, respectively. Asterisks represent significant differences from the controls obtained using one-way ANOVA with Tukey's test or the nonparametric Kruskal–Wallis test with Dunn's test. *p < 0.05, **p < 0.01, ***p < 0.001, NS not significant.

Species	Organ	Cd level	Cd mg kg^{-1}	N m/m%	P m/m%	K m/m%	Ca m/m%	Cu mg kg^{-1}	Mg m/m%	Fe mg kg^{-1}	Zn mg kg^{-1}
Maize	Shoot	Cd0	1.90 ±0.41	3.04 ±10.05	0.212 ±0.013	3.92 ±0.26	0.306 ±0.007	17.9 ±1.7	0.431 ±0.019	177.3 ±7.8	10.73 ±0.67
		Cd20	14.33 ±0.83***	2.57 ±0.15**	0.176 ±0.011*	3.43 ±0.09*	0.322 ±0.004^{NS}	17.2 ±0.8^{NS}	0.456 ±0.032^{NS}	142.0 ±15.9^{NS}	8.00 ±0.87*
		Cd50	17.94 ±1.69***	2.56 ±0.16**	0.171 ±0.011*	3.35 ±0.09*	0.285 ±0.020^{NS}	17.2 ±1.0^{NS}	0.421 ±0.020^{NS}	205.3 ±44.1^{NS}	8.58 ±0.79*
	Root	Cd0	2.20 ±0.47	2.60 ±0.19	0.091 ±0.013	2.35 ±0.010	0.349 ±0.036	80.6 ±1.9	1.280 ±0.056	1930 ±217	15.93 ±1.74
		Cd20	109.1 ±15.4***	2.66 ±0.23^{NS}	0.085 ±0.008^{NS}	2.27 ±0.09^{NS}	0.332 ±0.020^{NS}	77.2 ±3.2^{NS}	1.087 ±0.339^{NS}	1847 ±284^{NS}	19.70 ±2.33^{NS}
		Cd50	139.8 ±19.4***	2.59 ±0.20^{NS}	0.083 ±0.010^{NS}	2.17 ±0.26^{NS}	0.353 ±0.012^{NS}	72.9 ±6.6^{NS}	1.290 ±0.266^{NS}	2347 ±261^{NS}	18.23 ±2.80^{NS}
Cucumber	Shoot	Cd0	1.57 ±0.30	2.70 ±0.14	0.341 ±0.056	1.98 ±0.19	1.893 ±0.042	20.8 ±2.3	1.653 ±0.180	136.1 ±23.0	10.59 ±1.16
		Cd20	27.90 ±2.40***	2.63 ±0.09^{NS}	0.357 ±0.023^{NS}	1.86 ±0.11^{NS}	1.587 ±0.081**	19.5 ±2.0^{NS}	1.717 ±0.117^{NS}	83.6 ±8.8*	6.41 ±1.22*
		Cd50	38.13 ±3.52***	2.62 0.16^{NS}	0.329 ±0.012^{NS}	1.81 ±0.14^{NS}	1.330 ±0.026***	15.9 ±1.3*	1.760 ±0.044^{NS}	92.6 ±9.9*	6.25 ±1.19*
	Root	Cd0	4.98 ±0.81	2.60 ±0.19	0.161 ±0.011	2.49 ±0.24	0.574 ±0.064	104.0 ±4.0	1.527 ±0.055	3047 ±153	90.2 ±12.0
		Cd20	604 ±47.4***	2.88 ±0.20^{NS}	0.171 ±0.015^{NS}	2.38 ±0.12^{NS}	0.521 ±0.018^{NS}	96.8 ±4.2^{NS}	1.323 ±0.116^{NS}	2187 ±165**	94.5 ±23.1^{NS}
		Cd50	889.7 ±54.3***	2.79 ±0.20^{NS}	0.158 ±0.007^{NS}	2.22 ±0.18^{NS}	0.469 ±0.015*	86.8 ±6.3*	1.337 ±0.219^{NS}	2503 ±196*	102.0 ±20.8^{NS}
Pea	Shoot	Cd0	1.75 ±0.29	3.31 ±0.13	0.243 ±0.028	1.67 ±0.04	0.954 ±0.081	11.2 ±1.2	0.742 ±0.048	68.9 ±7.0	7.74 ±0.55
		Cd20	15.33 ±0.92***	2.57 ±0.18*	0.174 ±0.027*	1.41 ±0.05*	0.946 ±0.033^{NS}	8.4 ±0.8*	0.796 ±0.082^{NS}	64.4 ±2.5^{NS}	8.39 ±1.01^{NS}
		Cd50	33.63 ±2.42***	2.35 ±0.37**	0.182 ±0.010*	1.45 ±0.12*	0.972 ±0.050^{NS}	8.2 ±0.6*	0.821 ±0.023^{NS}	62.8 ±3.8^{NS}	5.52 ±0.67*
	Root	Cd0	2.37 ±0.33	3.28 ±0.11	0.168 ±0.008	3.10 ±0.13	0.495 ±0.032	103.9 ±7.4	0.891 ±0.072	1379 ±114	75.13 ±7.99
		Cd20	256.3 ±30.6***	2.62 ±0.14**	0.168 ±0.005^{NS}	2.52 ±0.09***	0.436 ±0.017*	73.3 ±7.6**	0.735 ±0.031*	935 ±74**	42.20 ±4.35***
		Cd50	470.4 ±28.4***	2.44 ±0.14***	0.178 ±0.006^{NS}	2.70 ±0.04**	0.390 ±0.014**	55.0 ±5.4***	0.685 ±0.009**	945 ±82**	40.57 ±2.06***

Table S2: Relative changes in the measured plant parameters compared to controls for maize, cucumber and pea exposed to 20 and 50 mg kg^{-1} substrate Cd levels (*Cd20* and *Cd50*, respectively) for 7 days. C_R – root electrical capacitance; Chl – leaf chlorophyll content; F_v/F_m – photosynthetic efficiency; g_s – stomatal conductance; SDM – shoot dry mass; RDM – root dry mass; RL – total root length.

Parameter	Maize		Cucumber		Pea	
	Cd20	Cd50	Cd20	Cd50	Cd20	Cd50
C_R	–3.9%	–15.9%	–6.7%	–16.2%	–10.5%	–35.9%
Chl	–5.0%	–9.4%	–7.1%	–13.5%	–40.4%	–62.0%
F_v/F_m	–1.3%	–0.6%	+0.5%	–0.1%	–22.4%	–65.4%
g_s	–18.2%	–25.4%	–21.7%	–35.6%	–52.3%	–81.7%
SDM	–0.6%	–4.8%	–3.5%	–15.9%	–16.9%	–40.6%
RDM	+0.2%	–13.3%	–14.0%	–22.8%	–19.0%	–37.1%
RL	–2.8%	–22.8%	–6.1%	–31.3%	–24.7%	–43.8%
